# Metastatic urothelial carcinoma^☆^^☆☆^

**DOI:** 10.1016/j.abd.2020.06.028

**Published:** 2021-05-24

**Authors:** Elisabeth Gómez-Moyano, Silvestre Martínez Garcia, David Hernandez Alcaraz, Maria Ayala-Blanca

**Affiliations:** Hospital Regional Universitario de Málaga, Malaga, Spain

Dear Editor,

A 61-year-old-man of the South of Spain presented at the Dermatology Department at Hospital Regional Universitario de Malaga with multiple painful erythemato-violaceous nodules on the chin, on the trunk, and the scalp (Fig. 1). The patient had been diagnosed with muscle-invasive micropapillary bladder cancer one year before and received treatment with radiotherapy and chemotherapy with cisplatin in a sparring bladder protocol treatment. A skin biopsy of the trunk was performed, showed medium-sized atypical cells arranged in nests or sheets, which exhibited round–ovoid nuclei and abundant eosinophilic cytoplasm, and they formed small rosette-like aggregates, in the superficial and deep dermis (Fig. 2). Immunohistochemical stains with CK-20 and CK-7 were positive.

**Figure 1:**
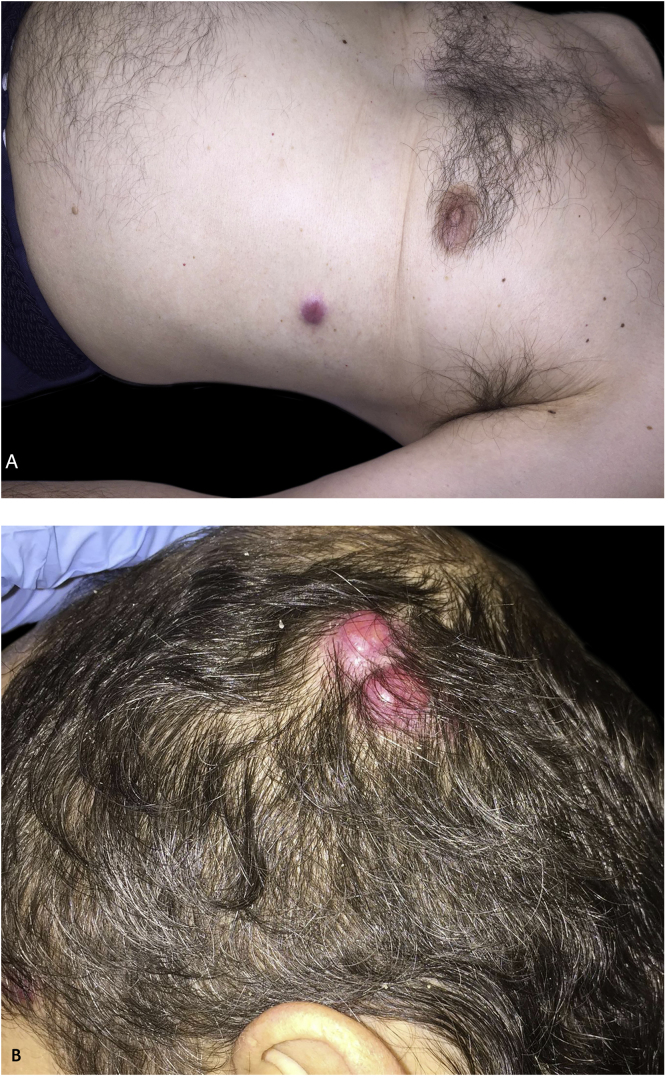
Multiple erythemato-violaceous nodules on the trunk (A) and the scalp (B).

**Figure 2:**
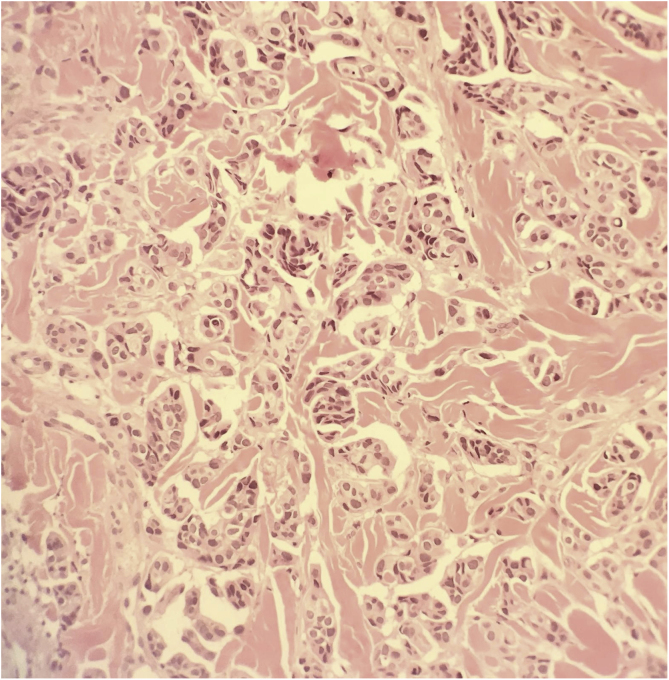
Medium-sized tumor cells arranged in nests (Hematoxylin & eosin, ×400).

The abdominal-pelvic CT image showed diffuse wall bladder thickening after therapy have been finished.

Multiple brain lesions compatible with metastasis were observed in the cranial MRI. Computerized tomography showed mediastinal lymph nodes, subcutaneous nodules, and pulmonary parenchyma infiltration compatible with carcinomatous lymphangitis. The scintigraphy image showed multiple bone metastases.

The patient started treatment with pembrolizumab, oral corticosteroids and holocranial radiotherapy (20 Gy), but he died seven months later.

The urothelial carcinomas are the second most common genitourinary tumors.^1^ The localized disease represents about 80% of cases, with the remainder presenting with regional or distant involvement.^1^ The most usual sites of metastases are the lymph nodes, liver, lung, bone, and rarely, brain metastases. Skin metastases from urothelial cancers are uncommon, with an incidence of 0.84%–3.6% and they are considered to be a poor prognostic sign. Metastatic infiltration of the skin may occur due to:^2^, ^3^ a) Direct tumor extension; b) Hematogenous or lymphatic spreading; c) Iatrogenic implantation of tumor cells.

The diagnosis requires a high index of clinical suspicion and histological confirmation.

The clinical presentation is quite diverse, and most commonly includes isolated nodular subcutaneous metastases. However, patients also present with diffuse, erythematous, raised infiltrates within the skin. Zosteriform pattern, extramammary Paget’s disease, have been documented. Erythema gyratum repens and acanthosis nigricans as paraneoplastic disorders have been reported.^4^, ^5^

A case of urothelial cutaneous metastasis mimicking condylomata acuminate has been reported. Penile metastasis from primary bladder cancer is an extremely rare event.^2^

Prognosis of patients with bladder cancer cutaneous spreading is generally poor with less than 1-year median survival.^3^

The optimal treatment of urothelial bladder cancer with micropapillary variant histology remains not clear.^4^ Although this tumor is associated with adverse clinicopathological features, a recent meta-analysis suggests that micropapillary urothelial bladder cancer does not necessarily mandate different treatment algorithms.^4^

Optimal management of patients with cT1 (tumor invades subepithelial connective tissue, clinical-stage) remains divergent. Each case should be discussed individually considering other clinicopathological factors and discuss management options as part of a shared decision-making process.^4^

## Financial support

None declared.

## Authors’ contributions

Elisabeth Gómez-Moyano: Data curation; formal analysis; investigation; methodology; project administration; software; supervision; validation; visualization; roles/writing – original draft; writing – review.

Silvestre Martínez Garcia: Formal analysis; investigation; methodology; project administration; software; supervision; validation; visualization; roles/writing – original draft; writing – review.

David Hernandez Alcaraz: Formal analysis; investigation; methodology; project administration; supervision validation; visualization; roles/writing – original draft; writing – review.

Maria Ayala-Blanca: Investigation; methodology; project administration; supervision; validation; visualization; roles/writing – original draft; writing – review.

## Conflicts of interest

None declared.

## References

[1] Raghavan D. (2016). Cutaneous manifestations of genitourinary malignancy. Semin Oncol.

[2] GiunchI F., Vasuri F., Valerio V., Montagnani I., Nelli F., Fiorentino M. (2017). Unusual asymptomatic presentation of bladder cancer metastatic to the penis. Pathol Res Pract.

[3] Kerkeni W., Ayari Y., Charfi L., Bouzouita A., Ayed H., Cherif M. (2017). Transitional bladder cell carcinoma spreading to the skin. Urol Case Rep.

[4] Abufaraj M., Foerster B., Schernhammer E., Moschini M., Kimura S., Hassler M. (2019). Micropapillary urothelial carcinoma of the bladder: a systematic review and meta-analysis of disease characteristics and treatment outcomes. Eur Urol.

[5] Thomaidou E., Armoni G., Klapholz L., Hadayer N., Maly A., Ramot R. (2018). Zosteriform cutaneous metastases. Clin Exp Dermatol.

